# Blocking podoplanin suppresses growth and pulmonary metastasis of human malignant melanoma

**DOI:** 10.1186/s12885-019-5808-9

**Published:** 2019-06-17

**Authors:** Mengqiao Xu, Xia Wang, Yanfang Pan, Xingpeng Zhao, Bin Yan, Changgeng Ruan, Lijun Xia, Yiming Zhao

**Affiliations:** 1 Jiangsu Institute of Hematology, Key Laboratory of Thrombosis and Hemostasis of the Ministry of Health, The First Affiliated Hospital of Soochow University, 188 Shizi Street, Suzhou, 215006 Jiangsu China; 2 Collaborative Innovation Center of Hematology, Soochow University, Suzhou, 215006 Jiangsu China; 3 The Affiliated Suzhou Hospital of Nanjing Medical University, Suzhou, 215006 Jiangsu China; 4 Drug Farm. Inc, Shanghai, 200000 China; 5 Clinical Laboratory Center, Luoyang Central Hospital Affiliated to Zhengzhou University, Luoyang, 471000 Henan China; 6 State Key Laboratory of Radiation Medicine and Protection, Soochow University, Suzhou, 215123 China

**Keywords:** Podoplanin, Antibody-based therapy, Malignant melanoma, Metastasis, Tumor growth

## Abstract

**Background:**

Podoplanin (PDPN), a transmembrane *O*-glycoprotein, is up-regulated in many tumors and is involved in tumor metastasis and malignant progression. In previous studies, we generated a functional blocking monoclonal antibody (mAb, SZ168) against the extracellular domain of human PDPN. This study is aimed to investigate whether blocking PDPN by SZ168 inhibits tumor growth and metastasis.

**Methods:**

Malignant melanoma xenograft model by inoculating subcutaneously human malignant melanoma cell line C8161 into the back of BALB/c nude mice was used. Endogenous PDPN expression in C8161 cells and nasopharyngeal cancer cell line CNE-2 was detected using western blot and flow cytometry.

**Results:**

SZ168 significantly inhibited C8161 or CNE-2 cell-induced platelet aggregation in a dose-dependent manner with a maximal inhibition of 73.9 ± 3.0% in C8161 cells or 77.1 ± 2.7% in CNE-2 cells. Moreover, SZ168 inhibited the growth and pulmonary metastasis of C8161cells in vivo. The number of lung metastatic foci in the SZ168-treated group was significantly decreased compared with that in the control mouse IgG group (1.61 ± 0.44 vs.3.83 ± 0.60, *P* < 0.01). Subcutaneous tumor volume, weight, and incidence were also significantly reduced in the SZ168-treated group compared to the control group (*P* < 0.05). Additionally, SZ168 recognized PDPN in immunohistochemical analyses of tumor tissue sections.

**Conclusions:**

SZ168 blocks growth and pulmonary metastasis of human malignant melanoma by inhibiting the interaction between tumor PDPN and platelet CLEC-2 and therefore is a promising antibody for therapeutic development against malignant melanoma.

## Background

Tumor growth and metastasis are highly complex processes that are affected by a wide variety of factors. Extensive evidence suggests that platelets play a key role in tumor cell proliferation and metastasis [1, 2]. One of the mechanisms is tumor cell-induced platelet aggregation (TCIPA) [3, 4], which may enhance embolism in the microvasculature and prevents elimination by host immune system.

Podoplanin (PDPN) is a transmembrane sialo-glycoprotein and its overexpression has been detected in many types of tumors, including squamous cell carcinoma [5–7], malignant mesothelioma [8, 9], Kaposi’s sarcoma [10], testicular seminoma [11], and brain tumors [12]. Recent studies suggested that the role of PDPN is associated with tumor metastasis, malignancy, and poor prognosis [13–18]. The extracellular domain of PDPN contains a heavily glycosylated amino terminal of approximately 130 amino acids, and conserved amino acid sequence EDXXVTPG is designated as the platelet aggregation stimulating (PLAG) domain [19]. PDPN is the only known endogenous ligand of the C-type lectin-like receptor 2 (CLEC-2) expressed on platelets [20]. The binding of tumor cell PDPN to platelet CLEC-2 triggers platelet activation and aggregation [21, 22]. To date, a number of anti-human PDPN monoclonal antibodies (mAbs) have been established; however, other than the rat anti-hPDPN mAb NZ-1 and a few mAbs that inhibit PDPN-induced platelet aggregation [23], most fail to block the interaction between PDPN and CLEC-2.

We have produced mAbs (SZ163 and SZ168) against the extracellular domain of human PDPN, and both exhibited high specificity and sensitivity [24]. An SZ163/SZ168-double-antibody sandwich enzyme-linked immunosorbent assay (ELISA) was developed to quantitate plasma-soluble PDPN in cancer patients and evaluate the correlation between PDPN and tumor occurrence and metastasis [24], although it is unknown whether SZ163 and SZ168 inhibit the growth and metastases in PDPN-expressing human tumors.

In this study, we showed that SZ168 inhibited platelet aggregation induced by PDPN-expressing human cancer cells in a dose-dependent manner. Furthermore, we found that SZ168 inhibited tumor growth and suppresses pulmonary metastasis in PDPN-expressing tumors in vivo.

## Methods

### Mice

Female BALB/c nude mice (4–5 weeks old) were purchased from Shanghai SLRC Experimental Animal Co. Ltd. (Shanghai, China) and maintained under specific pathogen-free conditions. Compressed CO2 asphyxiation was used to sacrifice mice in accordance with the recommendations of the Panel on Euthanasia of the American Veterinary Medical Association. All animal procedures were approved by the Animal Use and Ethics Committee of Soochow University (Suzhou, China).

### Cell lines

The Chinese hamster ovary (CHO) cell lines, nasopharyngeal carcinoma cells line CNE-2, and C8161 melanoma cell line were purchased from American Type Culture Collection (Gaithersburg, MD, USA). NCI-H226 human non-small cell lung tumor cell line was purchased from Jiangsu KeyGEN BioTECH Co. Ltd. (Nanjing, China). Mycoplasma Stain Assay Kit (Beyotime Institute of Biotechnology, Beijing, China) was used for testing mycoplasma contamination. None of the cell cultures were contaminated with mycoplasma. CHO cells expressing human podoplanin (CHO/hPDPN) were established as described previously [25]. CHO/hPDPN and C8161 cells were cultured in Dulbecco’s Modified Eagle’s Medium (DMEM; Hyclone, Logan, UT, USA), supplemented with 10% heat-inactivated fetal bovine serum (FBS; Gibco, Carlsbad, CA, USA). CNE-2 and NCI-H226 cells were cultured in RPMI 1640 medium (HyClone), supplemented with 10% FBS. These cell lines were cultured at 37 °C in a humidified atmosphere of 5% CO_2_. All human materials related studies were approved the Ethics Committee of the First Affiliated Hospital of Soochow University.

### Antibodies

SZ163 and SZ168, two mouse anti-hPDPN mAbs, were developed as described previously [24]. A mouse anti-hPDPN mAb (18H5), a normal mouse IgG (ab188776), and a rabbit anti-hPDPN mAb (EPR7072) were purchased from Abcam (Cambridge, UK). A mouse beta-actin antibody was purchased from ProteinTech (Wuhan, China). Fluorescein isothiocyanate-conjugated goat anti-mouse IgG polyclonal antibodies (FITC-GAM IgG) were purchased from Beckman-Coulter (Suzhou, China).

### Flow cytometry

Flow cytometry was performed as previously described [24]. Cultured cells were harvested by brief exposure to trypsin-ethylenediaminetetraacetic acid (EDTA) treatment and then incubated with 18H5 as a positive control, mouse IgG as a negative control, or anti-PDPN antibodies (SZ163 and SZ168, 2 μg) for 30 min at room temperature, followed by FITC-GAM IgG as secondary antibody for 30 min at room temperature. Flow cytometry was performed using a Cytomics FC500 machine (Beckman Coulter, CA, USA).

### Western blot analysis

Cells were solubilized using RIPA lysis buffer (Beyotime Biotechnology, Shanghai, China). The lysates were separated with 10% reduced SDS-PAGE and transferred onto a nitrocellulose membrane (Pall Corporation, New York, USA). After blocking with 5% skim milk in 0.1% PBST, the membrane was incubated with SZ163 or SZ168 (3 μg/mL), a rabbit anti-hPDPN mAb (EPR7072; 1:2000), or beta-actin antibody (1:2000) for 2 h at room temperature. Specifically-bound primary antibodies were detected with horseradish peroxidase-conjugated goat anti-mouse or goat anti-rabbit antibodies (1:10000, Immunotech, Marseille, France; 1:2000, Abcam, Cambridge, UK, respectively) and enhanced chemiluminescence (ECL) substrate (Sigma-Aldrich, St. Louis, MO, USA) according to the manufacturer’s instructions.

### Platelet aggregation assay

Human venous blood was collected from healthy donors in compliance with the Declaration of Helsinki, which was approved by the Ethics Committee of the First Affiliated Hospital of Soochow University. All participants gave written informed consent for this study. Platelet-rich plasma (PRP) was obtained from the whole blood supernatants by centrifugation at 100 x g for 10 min. The cells were harvested, washed, and resuspended in phosphate-buffered saline (PBS; 1 × 10^7^ cells/mL). For the antibody inhibition assay, cells were incubated with different concentrations of SZ163, SZ168, or control mouse IgG for 15 min on ice, followed by addition of 250 μL PRP. Platelet aggregation was measured in a Lumi-Aggregometer Model 700 (Chrono-log, Havertown, PA, USA). Data are provided as means ± SD of three independent experiments.

### In vivo lung metastasis model

CHO/hPDPN cells were harvested from culture using trypsin, washed, and resuspended in PBS (1 × 10^7^ cells/mL). The cells were incubated with SZ168 or mouse IgG and inoculated intravenously (1 × 10^6^ cells/mouse) into the lateral tail vein of (4–5 weeks old) female BALB/c nude mice. A total of 36 mice were used for three independent experiments, and each experiment involved 6 mice in the mouse IgG negative control group and 6 mice in the SZ168 treatment group. After 30 d, the mice were euthanized, and the number of lung surface metastatic foci was counted. The lungs and primary tumor tissues were also harvested for hematoxylin and eosin (H&E) staining.

### Immunohistochemical (IHC)

All tissue samples were fixed in formalin and embedded in paraffin, and 5-μm sections were cut out. After dewaxing, hydration, and antigen retrieval, sections were incubated with 2 μg/mL SZ168 or 18H5 overnight at 4 °C, followed by treatment with the Envision^+^ kit (MBX, Fuzhou, China) for 30 min and 3,3-diaminobenzidine tetrahydrochloride (DAB; MBX) for 1 min. Sections were subsequently counterstained with hematoxylin (MBX).

### Xenografts

CHO/hPDPN or C8161 cells were trypsinized, washed, and suspended with PBS (5 × 10^6^ cells/mL). The cells were inoculated subcutaneously into the backs of BALB/c nude mice (4–5 weeks old) at a dose of 200 μL per mouse. After 1 d, 30 μL of 1 mg/mL SZ168 or control mouse IgG were intravenously injected once a week for 3 weeks. Thirty-six mice were carried out for three independent experiments at different times, and each experiment was divided into mouse IgG negative control group mice and SZ168 treatment group, 6 mice per group. Tumor volumes were calculated every 3 d from caliper measurements of tumor dimensions using the formula (L × W^2^)/2, where L is the longer measurement. The mice were euthanized 27 or 30 d after tumor cell implantation. The lung tissues were harvested for H&E staining.

### Quantification of growth factors

C8161 cells (2.5 × 10^5^ cells/mL) were incubated with washed platelets (5 × 10^5^ platelets/mL) for 8 h at 37 °C in a 96-well culture plate (*n* = 6). After centrifuging at 3000 rpm for 5 min, the supernatants of the reaction mixtures were designated C8161-platelet reactants. The quantification of human growth factors, including PDGF and TGF-β-1, in the C8161-platelet reactants was conducted using enzyme-linked immunosorbent assays (ELISAs). All ELISA kits were purchased from R&D Systems (Wiesbaden, Germany) and were used according to the manufacturer’s instructions.

### Statistical analysis

Data are presented as the mean ± SD. Mann-Whitney U-test and two-way analysis of variance (ANOVA) were used to determine the statistical significance of the results in the tumor growth and metastasis models in vivo. *P < 0.05 was considered to be statistically significant. All statistical tests were two-sided.

## Results

### SZ163 and SZ168 detect PDPN expression on the membrane of C8161 and CNE-2 cells

**Fig. 1:**
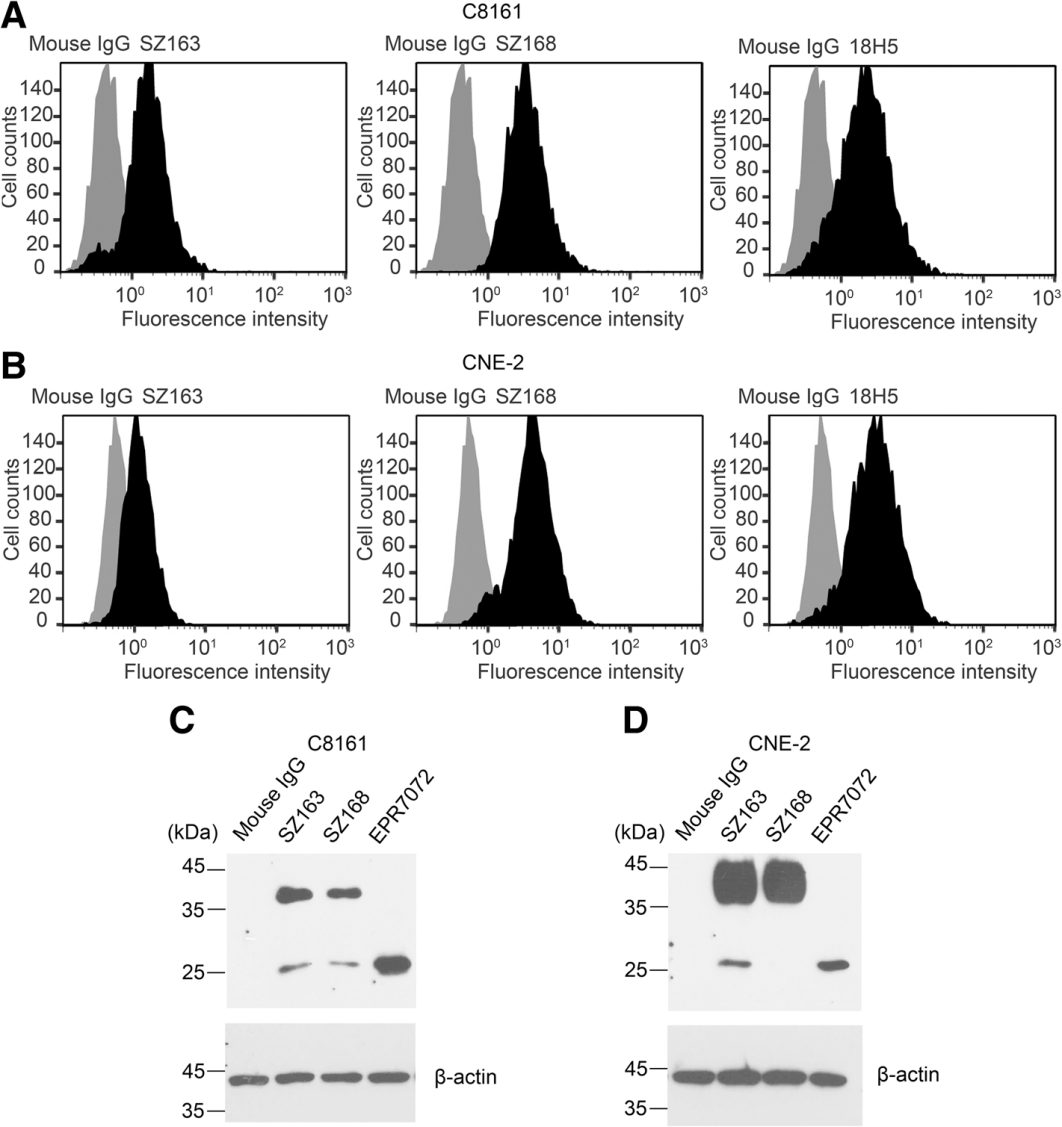
PDPN expression in human cancer cells is detected by mAbs SZ163 and SZ168. (**a**) C8161 or (**b**) CNE-2 cells were harvested and incubated with SZ163, SZ168, 18H5 (positive control), or mouse IgG (negative control), followed by incubation with FITC-goat anti-mouse antibody before flow cytometric analysis. The cells lysates of (**c**) C8161 or (**d**) CNE-2 were separated by 10% reduced SDS-PAGE and subjected to western blot using different anti-hPDPN mAbs as indicated, including SZ163and SZ168

Our previous study found that SZ163 and SZ168 detected hPDPN in CHO/hPDPN, NCI-H226, and U87 cells but not CHO cells [24], indicating that SZ163 and SZ168 specifically recognize hPDPN. Flow cytometry assay showed that SZ163 and SZ168 also detected endogenous PDPN in the human cancer cell lines C8161 and CNE-2, similarly to the 18H5 as positive control antibody (Fig. 1a, b). Additionally, western blot analysis revealed that both SZ163 and SZ168 bound to endogenous PDPN (36 or 25 kDa) in C8161 and CNE-2 cells (Fig. 1c, d).

### SZ168 inhibits platelet aggregation induced by human cancer cells with PDPN expression

**Fig. 2:**
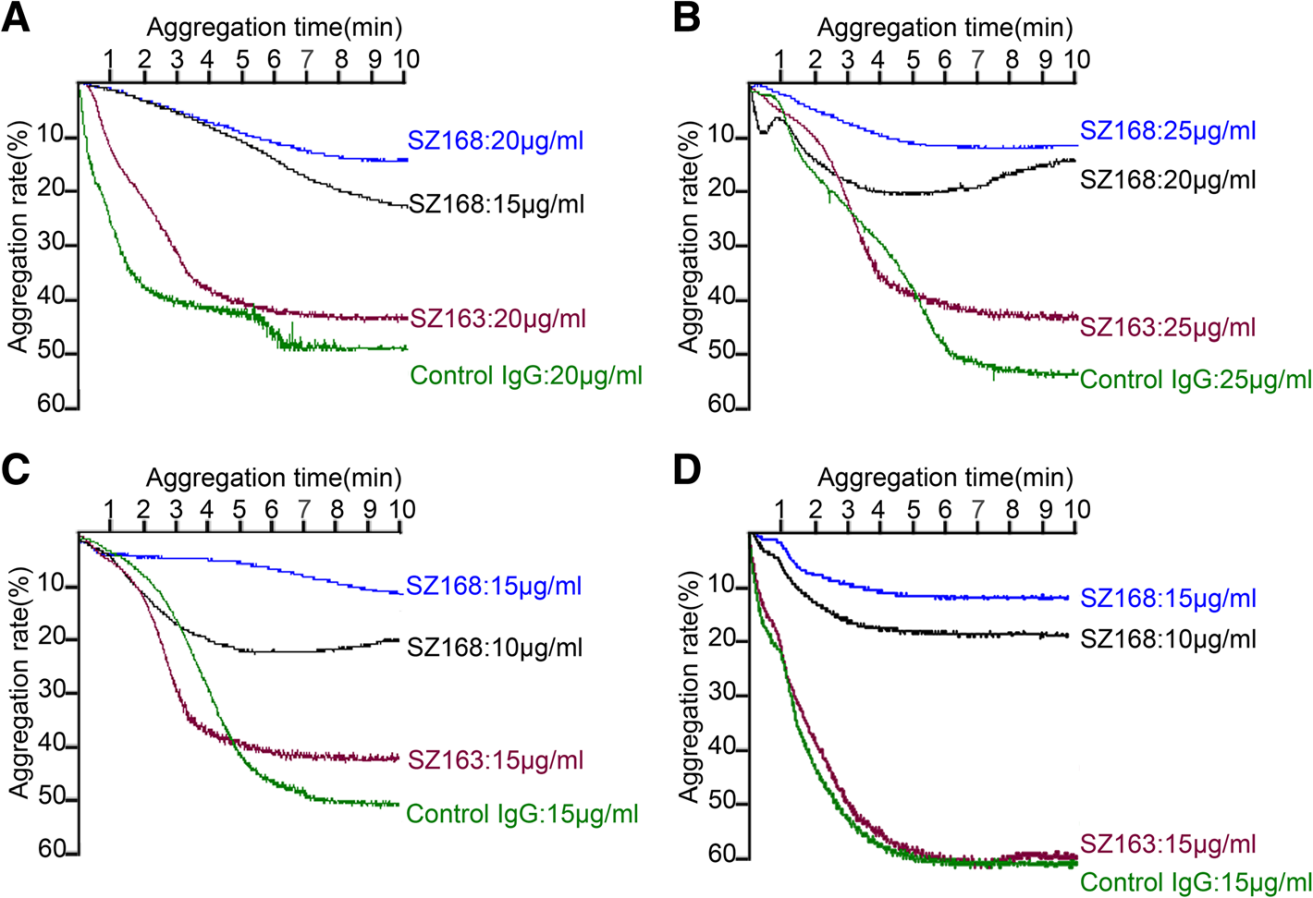
SZ168 inhibits platelet aggregation induced by human cancer cells with PDPN expression. (**a**) C8161 cells were pre-incubated with control mouse IgG (20 μg/mL), SZ168 (20, 15 μg/mL), or SZ163 (20 μg/mL). (**b**) CNE-2 cells were pre-incubated with control mouse IgG (25 μg/mL), SZ168 (25, 20 μg/mL), or SZ163 (25 μg/mL). (**c**) CHO/hPDPN and (**d**) NCI-H226 cells were pre-incubated with control mouse IgG (15 μg/mL), SZ168 (15, 10 μg/mL), or SZ163 (15 μg/mL). All cells were then incubated with 250 μL PRP to examine platelet aggregation in a lumi-aggregometer

Previously, Kato et al. showed that the PDPN-CLEC-2 interaction was essential for PDPN-induced platelet aggregation and that CHO/hPDPN cells but not CHO cells were able to induce platelet aggregation [19]. Therefore, we examined the ability of SZ163 and SZ168 to inhibit the platelet aggregation induced by C8161 or CNE-2 cells. Similarly, SZ168 significantly inhibited C8161- or CNE-2-induced platelet aggregation in a dose-dependent manner at 15, 20, and 25 μg/mL, whereas SZ163 did not (Fig. 2a, b). The maximal inhibition ratio of SZ168 was 73.9 ± 3.0% in C8161 cells at 20 μg/mL and 77.1 ± 2.7% in CNE-2 at 25 μg/mL of SZ168. Furthermore, SZ168 (15 μg/mL) also inhibited platelet aggregation induced by CHO/hPDPN and NCI-H226 cells with maximal inhibition ratios of 72.6 ± 3.4% and 74.2 ± 3.1%, respectively (Fig. 2c, d). Therefore, SZ168 is an anti-hPDPN mAb that inhibits platelet aggregation induced by tumor cells that express PDPN.

### SZ168 suppresses pulmonary metastasis in vivo

**Fig. 3:**
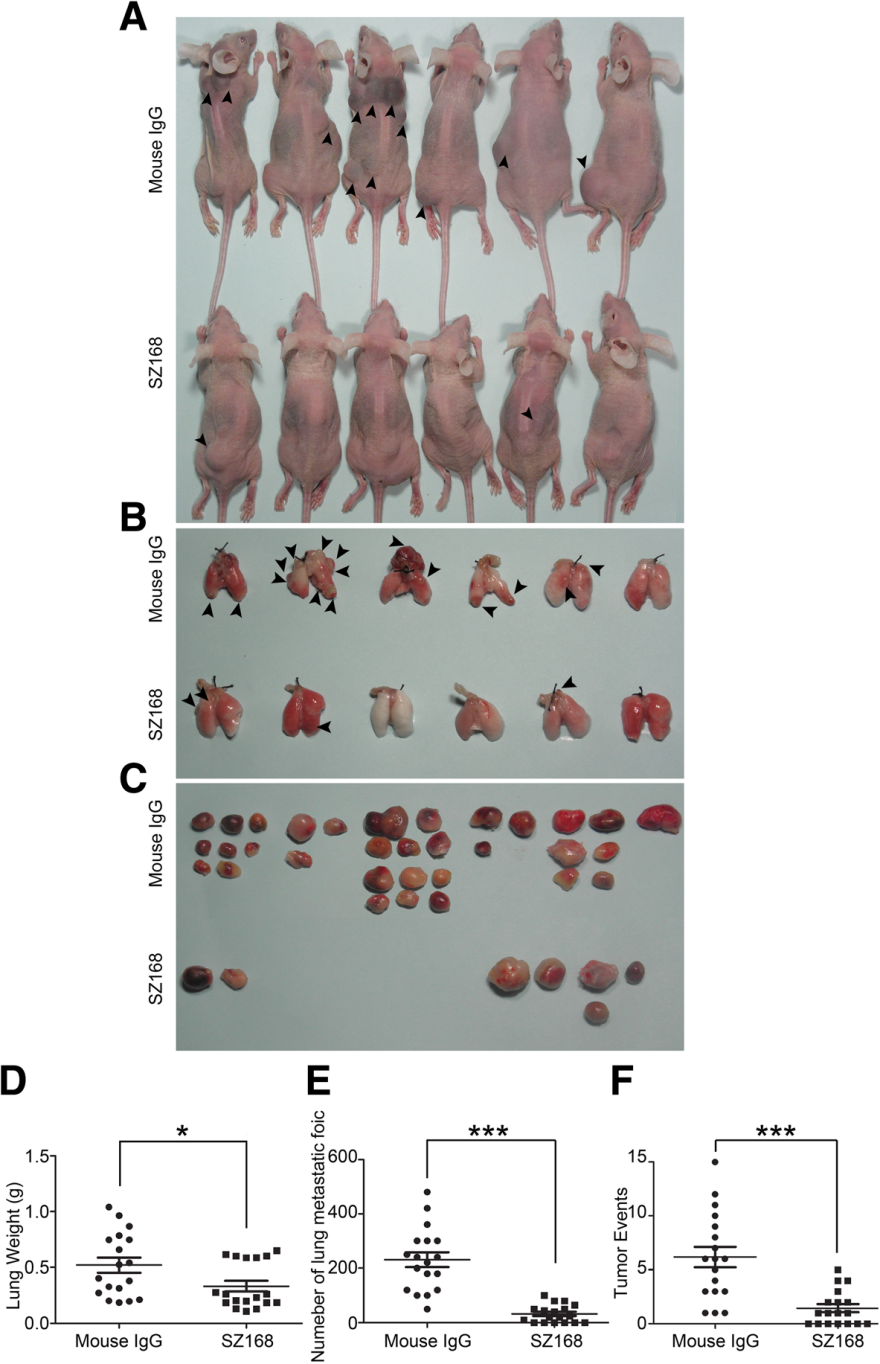
SZ168 suppresses pulmonary metastasis in vivo. CHO/hPDPN cells were incubated with 30 μg SZ168 or mouse IgG. Cell suspensions were intravenously inoculated into BALB/c nude mice. (**a**) Comparison of the tumor sizes and tumor incidences in mice on day 30. (**b**) Representative pictures of the lungs. (**c**) Gross morphology of the tumors. (**d**) Lung weights. *P < 0.05. (**e**) Numbers of metastatic foci of the lungs. ***P < 0.001. (**f**) Tumor events. ***P < 0.001. Arrows indicate subcutaneous tumors. Data are presented as mean ± SD from three independent experiments (*n* = 6)

**Fig. 4:**
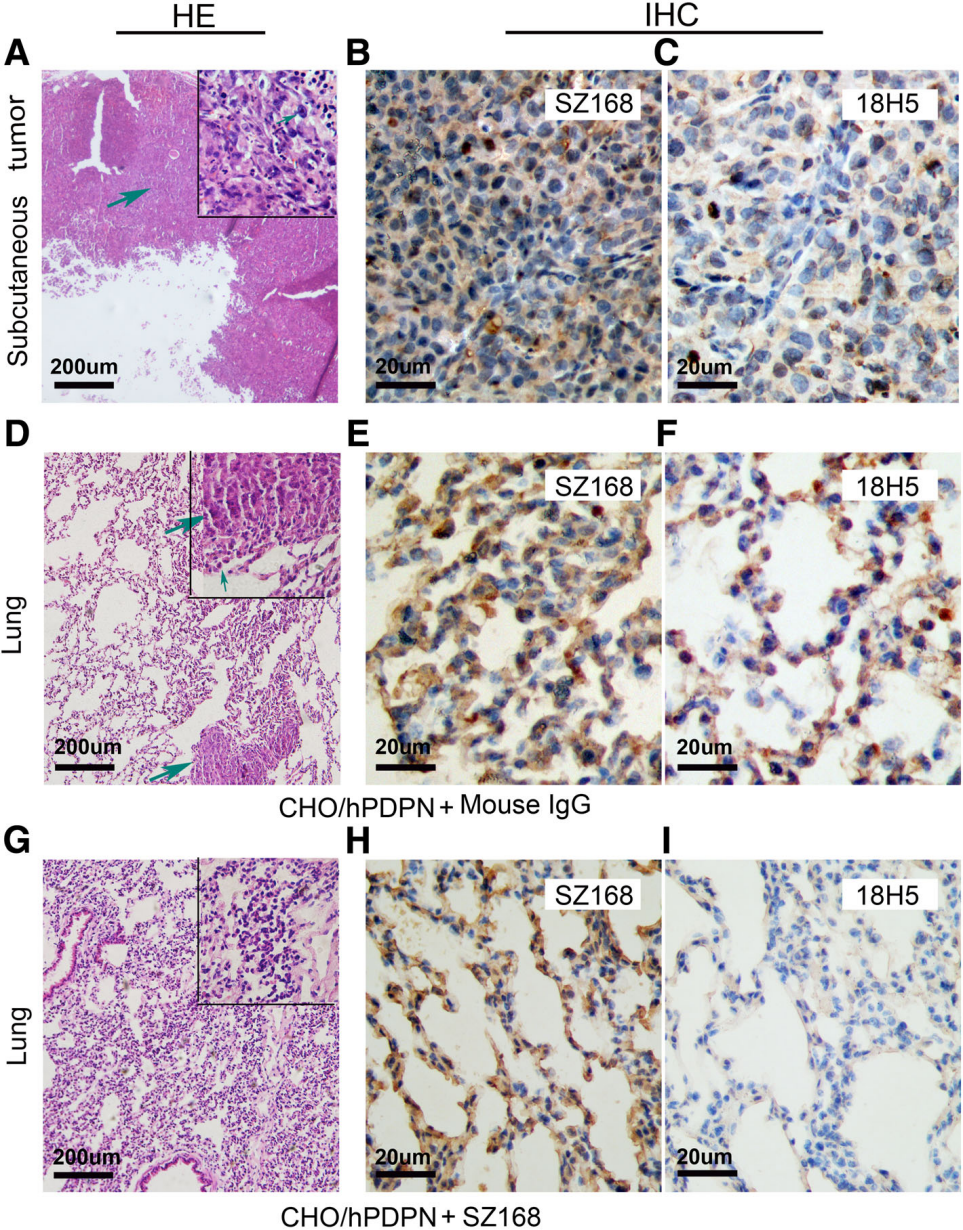
SZ168 detects hPDPN expression in the lung micro-metastasis foci and subcutaneous tumors in IHC analysis. The subcutaneous tumors (**a**) and lungs (**d, g**) were stained with H&E. Scale bar, 20 μm. Arrows indicate cancer nest. The subcutaneous tumors and lungs were incubated with SZ168 (**b, e, h**) or 18H5 (**c, f, i**) and then by the Envision^+^ kit reagents. Color was developed using DAB and subsequently counterstained with hematoxylin. Scale bar, 200 or 20 μm. Arrows indicate cancer nest

We next evaluated the effect of SZ168 on tumor growth and metastasis in vivo. Firstly, we examined whether SZ168 suppressed the pulmonary metastasis of intravenously-injected CHO/hPDPN cells. Although CHO cells have some metastatic potential, these cells become more metastatic following ectopic expression of PDPN [16]. The CHO/hPDPN cell-induced lung metastasis was nearly inhibited by administration of SZ168, and the number of metastatic foci (32.50 ± 5.70) was significantly lower than that of the control mouse IgG group (231.10 ± 27.25; *P* < 0.001) (Fig. 3b, e). Moreover, the number of tumor events in the mouse IgG-treated group (6.17 ± 0.95) was higher than that of the SZ168-treated group (1.44 ± 0.38; *P* < 0.001) (Fig. 3a, c, f). In addition, the weight of lungs was reduced in SZ168-treated mice (0.33 ± 0.05 g), compared to those in the control group (0.52 ± 0.07 g; *P* < 0.05) (Fig. 3d). Subcutaneous tumor formation was confirmed by H&E staining (Fig. 4a), and lung micro-metastasis foci and subcutaneous tumors were confirmed by immunostaining of PDPN using SZ168 (Fig. 4b, e, h) and 18H5 (Fig. 4c, f, i) antibodies. H&E staining revealed more metastatic foci in the lungs of the mouse IgG group than in those of the SZ168-treated group (Fig. 4d, h). These results indicated that SZ168 suppresses metastasis of CHO/hPDPN cells in both lung and subcutaneous tumors.

### SZ168 shows anti-tumor effect in CHO/hPDPN xenografts

**Fig. 5:**
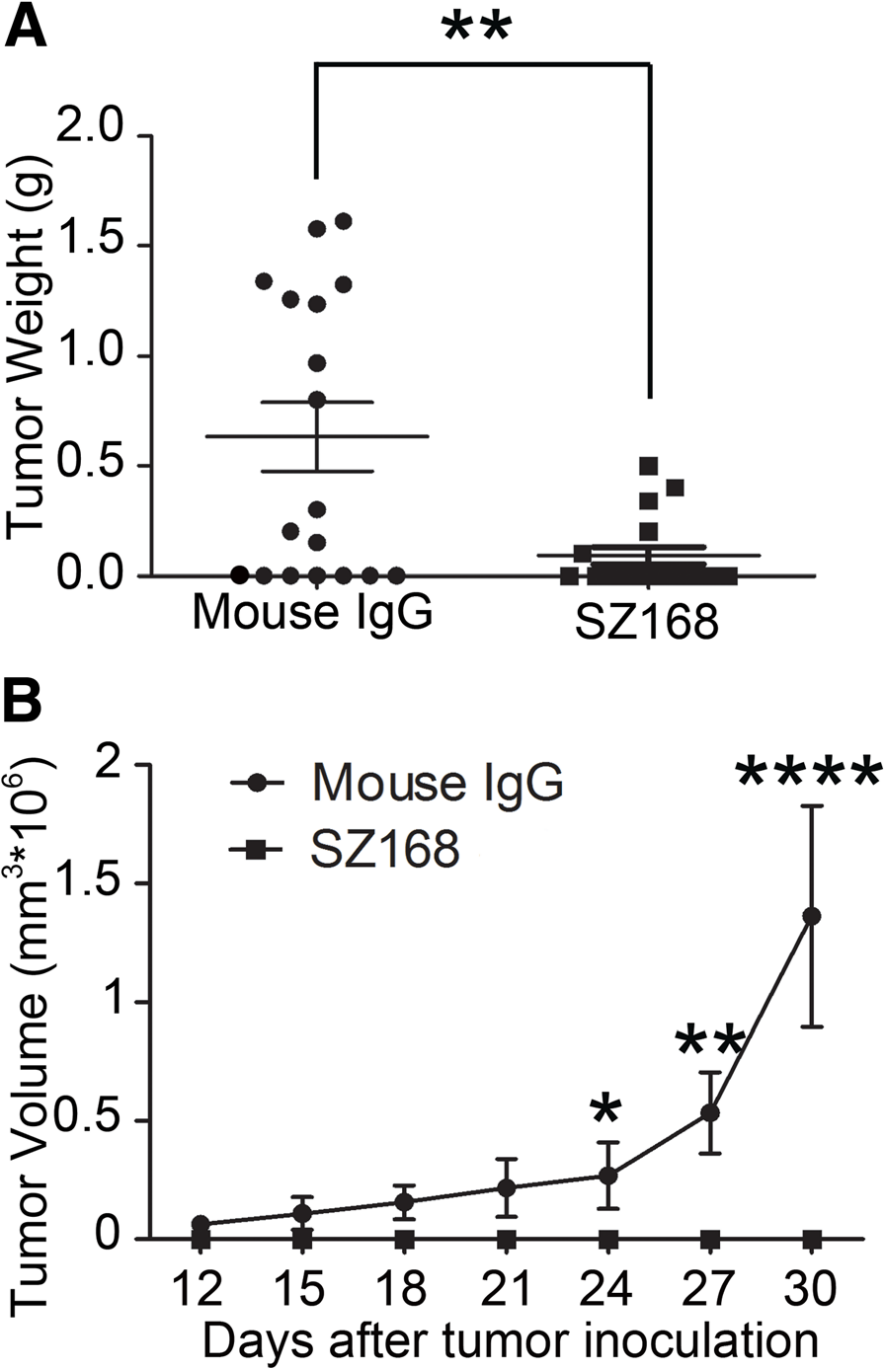
SZ168 inhibits tumor growth of CHO/hPDPN in vivo. CHO/hPDPN cells (1 × 10^6^ cells) were implanted into BALB/c nude mice. After 1 d, SZ168 or mouse IgG (30 μg/mouse) antibody was injected into the lateral tail vein of mice. (**a**) Primary tumor weights. **P < 0.01. (**b**) Primary tumor growth. Data are presented as mean ± SD from three independent experiments (*n* = 6)

A CHO/hPDPN xenograft model was used to further investigate the anti-tumor activity of SZ168 on primary tumor growth in vivo. The tumor formation was dramatically reduced in SZ168-treated group compared with that of control mouse IgG group (tumor incidence: 27.8% [5/18] vs. 61.1% [11/18]) (Fig. 5a). Meanwhile, tumor weight was significantly lower in the SZ168-treated group (0.09 ± 0.04 g) than in the control mouse IgG-treated group (0.63 ± 0.16 g; *P* < 0.01) (Fig. 5a). Tumor volumes were also significantly reduced by SZ168 treatment compared to IgG control treatment (Fig. 5b).

### SZ168 inhibits primary tumor growth and spontaneous pulmonary metastasis of C8161

**Fig. 6:**
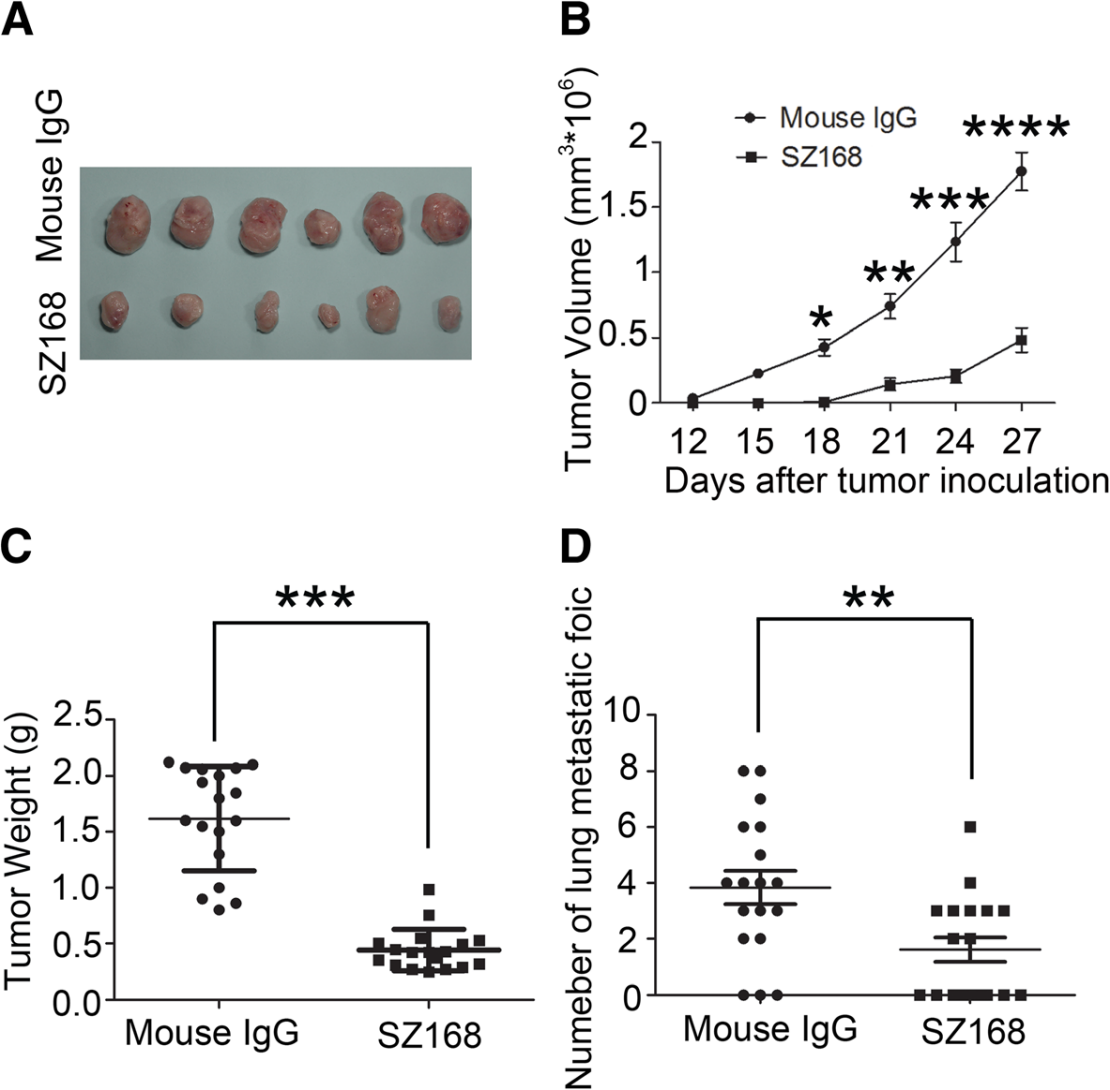
SZ168 restrains primary tumor development and spontaneous lung metastasis of C8161 cells in vivo. C8161 cells (1 × 10^6^ cells) were inoculated subcutaneously into BALB/c nude mice. After 1 d, SZ168 or mouse IgG (30 μg/mouse) antibody was injected into the lateral tail vein of mice. (**a**) Gross morphology of the tumor xenografts. (**b**) Primary tumor growth. (**c**) Primary tumor weights. ***P < 0.001. (**d**) Numbers of metastatic foci of the lungs. **P < 0.01. Data are presented as mean ± SD from three independent experiments (*n* = 6)

**Fig. 7:**
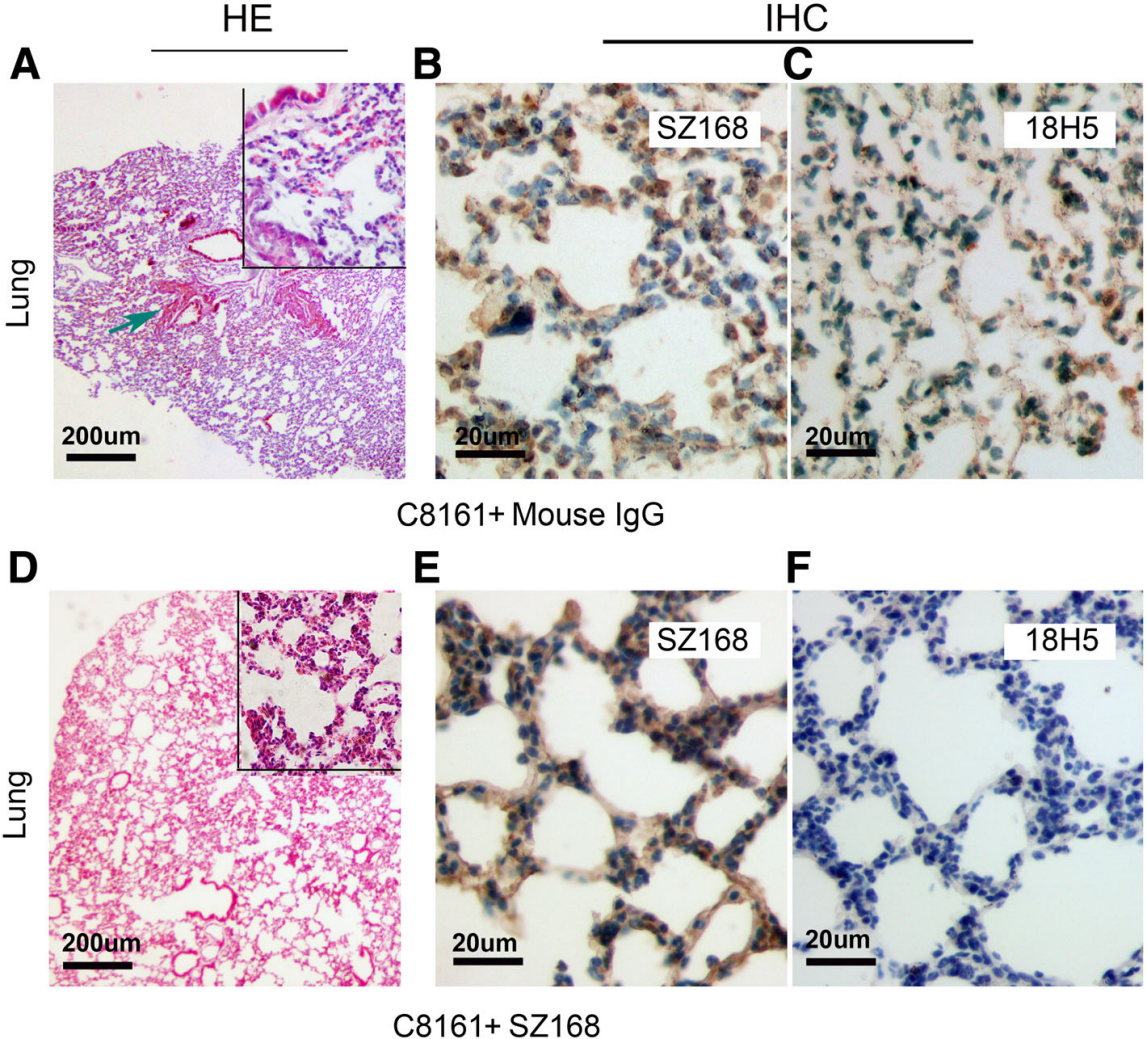
SZ168 detects hPDPN expression in the lung micro-metastasis foci by immunohistochemistry. Lung sections were stained with H&E (**a, d**). Scale bar, 200 μm. Arrows indicate cancer nest. Sections were incubated with (**b, e**) SZ168 or (**c, f**) 18H5 antibody as well followed by the Envision^+^ kit reagents. Color was developed using DAB and then counterstained with hematoxylin. Scale bar, 20 μm. Arrows indicate cancer nest

To investigate the anti-tumor effect of SZ168 on endogenous hPDPN-expressing cancer cells in vivo, C8161 cells were used in the established tumor model, as described above. Similarly, SZ168 dramatically reduced tumor development in these mice (Fig. 6a). In addition, tumor volumes and tumor weights were significantly reduced by SZ168 treatment compared to control IgG treatment (*P* < 0.05) (Fig. 6b, c). The number of metastatic foci in the SZ168-treated mice (1.61 ± 0.44) was significantly decreased compared to that in the control mouse IgG-treated mice (3.83 ± 0.60; *P* < 0.01) (Fig. 6d). Furthermore, H&E staining revealed that more metastatic foci were present in the lungs of the mice treated with mouse IgG than in those treated with SZ168 (Fig. 7a, d). Immunostaining with SZ168 (Fig. 7b, e) and 18H5 (Fig. 7c, f) detected PDPN and confirmed the presence of lung metastasis. Taken together, these results highlight the anti-tumor and anti-metastasis effects of SZ168.

### C8161 cells co-cultured with platelets induced the release of PDGF and TGFβ-1 from platelets

To reveal the contribution of the tumor-platelet interaction to the proliferation, transmigration, and invasion of C8161 cells, we measured the levels of platelet-derived growth factor PDGF-BB and transforming growth factor TGFβ-1 in the supernatants of C8161 cells co-cultured with platelets. As shown in Table 1, the concentrations of PDGF-BB and TGFβ-1 were notably higher in the group containing platelets (5 × 10^5^ platelets/mL) co-cultured with C8161 cells (2.5 × 10^5^ cells/mL) than in the negative control group (platelets alone) (*P < 0.05). Furthermore, after the C8161 group were pretreated with SZ-168, the concentrations of PDGF-BB and TGFβ-1 decreased compared to the negative control group (Mouse IgG) (**P < 0.05). These results suggest that the tumor-platelet interaction promotes the proliferation, migration, and invasion of C8161 cells, partly due to the interaction-induced release of PDGF-BB and TGFβ-1 by the platelets.

**Table 1:** C8161 cells induced the release of PDGF and TGFβ-1 from platelets

Groups	N	PDGF(pg/ml)	TGFβ-1(pg/ml)
Plts	6	148.6 ± 65.3	523.3 ± 183.4
Plts + C8161	6	445.7 ± 131.2*	2012.3 ± 315.8*
Plts + C8161 + SZ-168	6	216.2 ± 52.4**	751.8 ± 163.4**
Plts + C8161 + mouse IgG	6	472.8 ± 105.8	2156.4 ± 393.8

*P < 0.05, Plts+C8161 vs Plts

**P < 0.05, SZ-168 vs mouse IgG

## Discussion

Platelets have been reported to be involved in tumor cell-growth, metastasis, and invasiveness [26–28]. The following possible mechanisms have been proposed: (i) tumor cells are coated by platelets in the microvasculature and form large tumor cell-platelet aggregates, (ii) tumor cell-platelet aggregates protect tumor cells by forming a physical shield to protect tumor cells, and (iii) activated platelets release soluble factors that enhance tumor motility [29]. In addition, the administration of subcutaneous low molecular weight heparin has been found to prolong the survival of patients with advanced cancer [30]; however, administration of antiplatelet or anticoagulant drugs to patients receiving cancer therapy is risky due to bleeding concerns, particularly in patients’ chemotherapy-induced thrombocytopenia. Thus, it is desirable to have a novel targeted therapy to block platelet-cancer cell interactions.

The interaction between tumor cell PDPN and platelet CLEC-2 drives tyrosine phosphorylation of the Src family kinases (Syk) and phospholipase C gamma 2 (PLCγ2), resulting in platelet activation and aggregation [22, 31]. Activated platelets release secretory factors, such as transforming growth factor-β, vascular endothelial growth factor A, and platelet-derived growth factor, promoting tumor growth and angiogenesis [32, 33]. CLEC-2-deficient platelets had normal adhesion and spreading on platelet agonists except for a snake venom protein rhodocytin, indicating that the inhibition of PDPN-CLEC-2 interaction does not interfere with physiological hemostasis [31]. Thus, platelet-targeted therapy may be useful and biologically safe. PDPN is expressed in many types of tumors, as well as in normal tissues, including lymphatic vessels, type I alveolar epithelium, and kidney podocytes. The interaction between PDPN and CLEC-2 not only promotes cancer cell-induced platelet aggregation but also plays an essential role in physiological processes. PDPN knock-out mice exhibit impaired congenital lymphedema and lymphatic injury patterns [31]. Since cancer cells may interfere with physiological interaction between PDPN and CLEC-2, targeting the PDPN and CLEC-2 interaction seems to be a reasonable cancer treatment.

SZ163 and SZ168, two mAbs against human PDPN previously produced in our laboratory, showed high reactivity with PDPN-expressing cell lines CHO/hPDPN, U87, and NCI-H226. In the present study, we found that SZ163 and SZ168 specifically recognize endogenous hPDPN in cancer cell lines C8161 and CNE-2 by flow cytometry and western blot. Furthermore, comparison of the reactivity between SZ168 and 18H5 against hPDPN showed that SZ168 may be more sensitive and useful than 18H5 in flow cytometry. Moreover, SZ168 significantly inhibited platelet aggregation induced by C8161, CNE-2, NCI-H226, or CHO/hPDPN in a dose-dependent manner, whereas SZ163 IgG did not. Furthermore, SZ168 suppressed tumor growth and metastasis in CHO/hPDPN and C8161cells in vivo. Both tumor weight and the number of lung micro-metastasis foci were significantly lower in the mice treated with SZ168 than in those treated with control mouse IgG group. It is reported that NZ-1 and MS-1 inhibit PDPN-CLEC-2 interaction in vitro, but whether these antibodies suppress human cancer growth and/or metastasis remains unknown [23, 34]. We found that SZ168 not only suppresses PDPN-induced platelet aggregation in vitro but also inhibits tumor growth and metastasis in both mouse (CHO/hPDPN) and human (C8161) cancer cells. The experimental results confirmed that the PDGF and TGFβ-1 secreted by platelets can significantly promote the metastasis of tumor cells. Moreover, the migration-promoting effects of PDGF and TGF-β were partially inhibited by the mAb SZ-168. Furthermore, SZ168 may also be useful for diagnostics in immuno-histochemical analysis due to its high sensitivity, similar to another anti-PDPN antibody, D2–40, which has been used as a marker for the diagnosis of many different human tumors by immunohistochemical analysis [5, 35].

Our data show that the growth of C8161 malignant melanoma is regulated via PDPN-CLEC-2-mediated platelet aggregation. Thus, SZ168 may suppress the growth of malignant melanoma cells in vivo by inhibiting platelet activation and reducing secretion of tumor growth factors. PDPN is up-regulated in skin melanoma, which is an aggressive tumor with an increasing incidence, a high degree of malignancy, and high metastatic rate [36, 37]. Notably, distant metastases in melanoma patients are associated with a lower five-year survival rate [38]. Our results indicate that SZ168 may be a promising antibody to be developed as targeted therapy for PDPN-expressing malignant melanoma.

## Conclusions

Our study shows that SZ168 is a blocking antibody to human PDPN that inhibits the interaction between tumor PDPN and platelet CLEC-2. Our results indicate that SZ168 antibody has high potential for the development of novel antibody-based immunotherapy against PDPN-expressing tumors such as human malignant melanoma.

## Data Availability

All data and materials are presented in the manuscript, which will be freely available to any scientists wishing to use them for non-commercial purposes.

## Abbreviations

CHO: Chinese hamster ovary
CHO/hPDPN: CHO cells expressing human podoplanin
CLEC-2: C-type lectin-like receptor 2
ECL: Chemiluminescence
EDTA: Ethylenediaminetetraacetic acid
ELISA: Enzyme-linked immunosorbent assay
FITC-GAM IgG: Fluorescein isothiocyanate-conjugated goat anti-mouse IgG polyclonal antibody
H&E: Hematoxylin and eosin
IHC: Immunohistochemical
mAb: Monoclonal antibody
PBS: Phosphate-buffered saline
PDPN: Podoplanin
PLAG: Platelet aggregation stimulating
PLCγ2: Phospholipase C gamma 2
PRP: Platelet-rich plasma
Syk: Src family kinases
TCIPA: Tumor cell-induced platelet aggregation
